# Proton arc therapy reduces the dose delivered to circulating blood compared to intensity‐modulated proton therapy for head‐and‐neck cancer

**DOI:** 10.1002/acm2.70864

**Published:** 2026-10-20

**Authors:** Sebastian Tattenberg, Peilin Liu, Xiaoda Cong, Cornelia Hoehr, Xuanfeng Ding

**Affiliations:** ^1^ Division of Life Sciences TRIUMF Vancouver British Columbia Canada; ^2^ Department of Radiation Oncology William Beaumont University Hospital, Corewell Health Royal Oak Michigan USA

**Keywords:** blood dose, proton arc therapy, proton therapy, radiation‐induced lymphopenia

## Abstract

**Background:**

Compared to intensity‐modulated proton therapy (IMPT), proton arc therapy (PAT) has various dosimetric advantages. However, questions have been raised over whether the larger low‐dose bath during PAT may increase radiation‐induced lymphocyte killing, which is correlated with adverse clinical outcomes.

**Purpose:**

The purpose of this study is to quantitatively compare radiation dose delivery to circulating blood during IMPT and PAT.

**Methods:**

For each of 10 head‐and‐neck cancer patients, one IMPT and one PAT treatment plan were created, and the hematological dose (HEDOS) blood dose model was used to simulate radiation dose delivery to circulating blood. HEDOS simulations utilized gantry angle‐wise dose distributions (including for PAT) and considered gantry rotation and energy layer switching as well as irradiation times. Blood dose histograms as well as the mean dose to all simulation blood particles (D_mean_) and the dose delivered to the 90% and 10% of blood particles receiving the highest dose (D_90%_ and D_10%_) were then compared between IMPT and PAT.

**Results:**

For IMPT, blood dose metrics (in the form of mean over all patients ± one standard deviation) were (0.522 ± 0.204)Gy(RBE) for D_90%_, (0.563 ± 0.212)Gy(RBE) for D_mean_, and (0.605 ± 0.219)Gy(RBE) for D_10%_. For PAT, D_90%_ was (0.430 ± 0.184)Gy(RBE), D_mean_ was (0.483 ± 0.195)Gy(RBE), and D_10%_ was (0.538 ± 0.207)Gy(RBE). The Wilcoxon signed‐rank test indicated statistically significant (*p <* 0.05) differences between blood dose metrics of IMPT and PAT for all patients, and IMPT did not increase the percentage of blood particles which received no radiation dose during the delivery of a single treatment fraction.

**Conclusions:**

Compared to IMPT, proton arc therapy was associated with significantly lower radiation dose delivery to circulating blood across a variety of different metrics. Proton arc therapy for head‐and‐neck cancer is therefore not expected to result in higher radiation‐induced killing of circulating lymphocytes compared to IMPT.

## 1 INTRODUCTION

Current clinical intensity‐modulated proton therapy (IMPT) for cancer utilizes target irradiation from just a few different angles, but proton arc therapy (PAT), for which the target is instead irradiated from at least one arc trajectory, has been reported to benefit from a variety of dosimetric advantages.^1^, ^2^, ^3^, ^4^ Despite the technical challenges of delivering PAT in a clinical context, technological progress has been made in recent years, and the first patients have now received proton arc therapy treatments.^5^, ^6^, ^7^ Even so, questions have previously been raised over whether the larger low‐dose bath associated with PAT may increase radiation‐induced lymphocyte killing, which has been reported to be correlated with adverse clinical outcomes such as lower patient survival.^8^, ^9^, ^10^, ^11^

A variety of models to simulate radiation dose delivery to circulating blood during radiotherapy have been developed, and blood dose estimates from such models have previously been reported to be correlated with higher radiation‐induced lymphopenia and adverse clinical outcomes.^12^, ^13^, ^14^, ^15^ However, to the best of our knowledge, such models have not yet been applied to proton arc therapy treatment plans. For models which include the simulation of the blood flow during treatment delivery, part of the reason for this may be the relative complexity of PAT treatment delivery, and the arising difficulties of accurately accounting for irradiation from numerous gantry positions as well as dynamic considerations such as energy layer switching times.

The purpose of this work is to determine the potential impact of PAT implementation on the radiation dose delivered to circulating blood for patients with head‐and‐neck cancer. For this purpose, an IMPT and a PAT treatment plan with consistent target coverage were created for each patient included in this study, and dynamic simulations of radiation delivery to circulating blood, which considered a wide range of treatment delivery mechanisms, were performed in order to compare dose delivery to circulating blood during PAT to the current clinical standard of IMPT.

## 2 METHODS

The dataset consisted of a total of 10 patients with head‐and‐neck squamous cell carcinoma (HNSCC). For each patient, an IMPT and a PAT treatment plan were created, relevant blood compartments which had not already been manually delineated on the treatment planning computed tomography (CT) scan were segmented automatically, and dynamic blood dose simulations of both treatment plans were performed. Subsequently, a variety of blood dose metrics were compared in order to determine the impact of PAT implementation on the radiation dose delivered to circulating blood, motivated by its implications for associated radiation‐induced lymphopenia.

### 2.1 Patient selection and treatment planning

A total of 10 patients were randomly selected from the “HNSCC‐3DCT‐RT” as well as the “Head‐Neck‐PET‐CT” dataset, both of which are publicly available in the Cancer Imaging Archive.^16^, ^17^, ^18^ An overview over a variety of patient characteristics is shown in Table 1. For every patient, one IMPT and one PAT treatment plan were created in Version 2023B of RayStation (RaySearch Laboratories, Stockholm, Sweden) using the beam model of an IBA ProteusONE (Ion Beam Applications, Louvain‐la‐Neuve, Belgium). All treatment planning utilized clinical target and organ‐at‐risk (OAR) constraints and objectives as well as worst‐case robust optimization, with a setup uncertainty of 3 mm, a proton range uncertainty of 3.5%, and 21 scenarios being included in the optimization. As is current clinical practice for patients like the ones in question, IMPT generally utilized four different gantry angles (60°, 160°, 220°, and 300°). The only exception to this was patient 8, for whom three gantry angles (40°, 90°, 160°) were used, in accordance with clinical practice at our institution in this case. Spot‐scanning proton arc therapy (SPArc) treatment plans consisted of a single arc with a sampling frequency of 2.5°.^19^ Each SPArc treatment plan underwent the process of control point re‐sampling, energy layer re‐distribution, energy layer filtration, and energy layer re‐distribution. The tolerance window setting was ± 1°, the calculation grid setting for all treatment plans was 3 mm*3 mm*3 mm, and the spot spacing setting was the default value also used for clinical treatment planning. Dose calculations were Monte Carlo simulation‐based throughout, with a statistical uncertainty setting of 0.5%. For a single patient, delineated structures as well as the dose distribution associated with the generated IMPT and PAT treatment plans are shown in Figure 1.

**TABLE 1:** The sex, age, primary site, and target volume, and radiotherapy prescription dose for all patients included in this study. D_HR_ refers to the prescription dose of the high‐risk target volume, since patients may have a number of different target volumes with varying prescription doses.

Patient	Sex	Age [years]	Primary site	Target volume [cm^3^]	D_HR_ [Gy(RBE)]	Chemo
**1**	Male	66	Base of tongue	488.1	70.0	Yes
**2**	Male	52	Tonsil	467.1	60.0	Yes
**3**	Male	87	Base of tongue	345.4	74.0	None
**4**	Female	55	Oral tongue	316.2	74.0	Yes
**5**	Male	69	Mandible	320.8	74.0	Yes
**6**	Male	66	Oropharynx	341.1	70.0	Yes
**7**	Male	71	Lymph nodes	424.1	66.0	Yes
**8**	Male	68	Occipital scalp	489.7	70.0	Yes
**9**	Male	80	Hard palate	226.4	60.0	None
**10**	Female	57	Unknown	320.7	70.0	Yes

**FIGURE 1:**
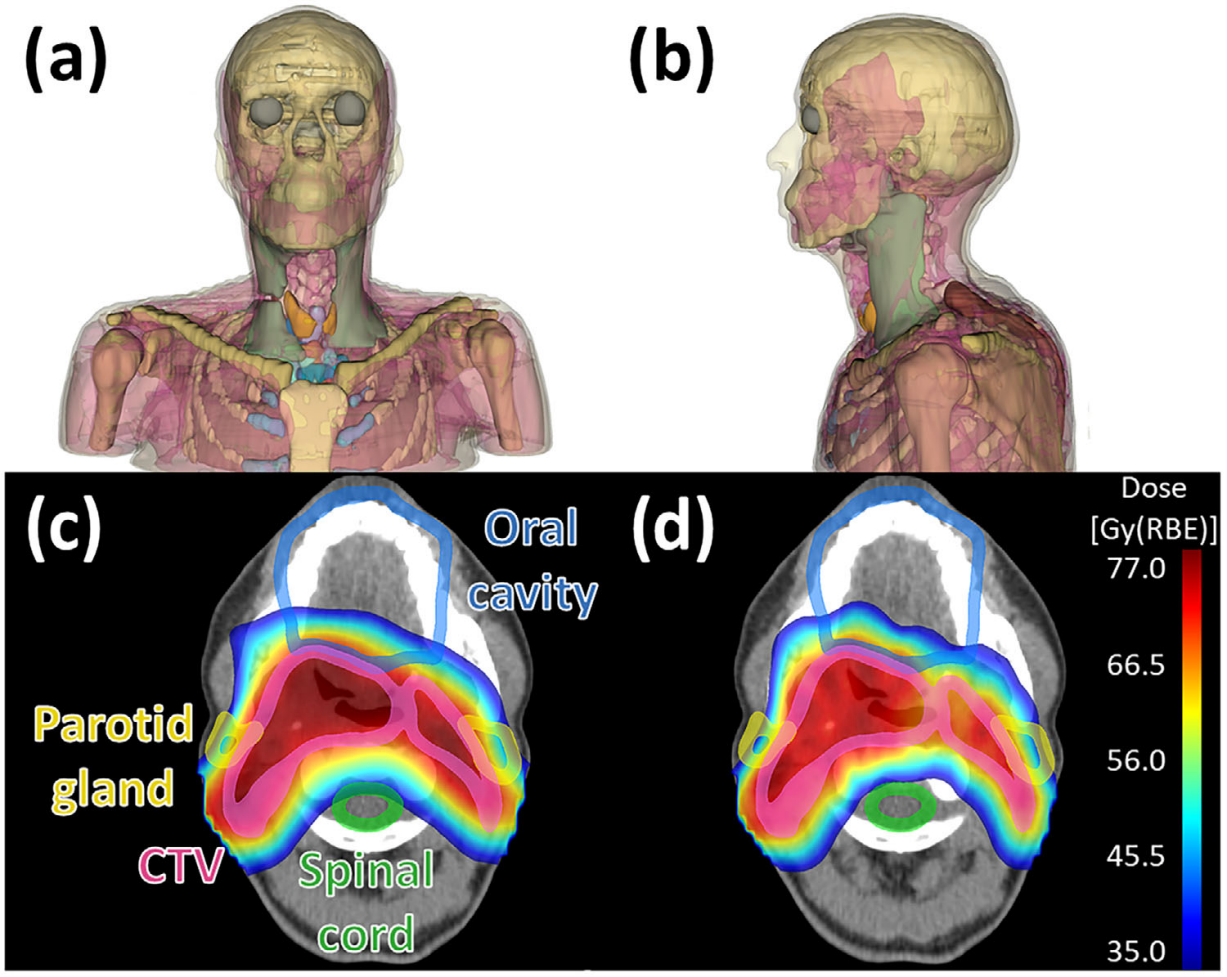
The delineated structures for one patient in (a) frontal and (b) side view as well as the dose distribution resulting from the generated (c) intensity‐modulated proton therapy (IMPT) and (d) proton arc therapy (PAT) treatment plan. The clinical target volume (CTV) is shown in pink, and the oral cavity, spinal cord, and parotid glands are depicted in blue, green, and yellow, respectively.

### 2.2 Auto‐contouring and blood dose simulations

Subsequently to treatment planning, TotalSegmentator was used to automatically delineate relevant OARs, blood vessels, and tissues which had not already been provided as part of the manually‐delineated structures included in the patient datasets.^15^, ^20^ In order to evaluate the potential impact of the distance between clinical target volume (CTV) and major blood vessels on simulated blood dose values, 95^th^ percentile Hausdorff distances between CTV and major blood vessels (HD_95CTV‐BV_) were determined using Plastimatch.^21^ The open‐source dynamic hematological dose (HEDOS) model, which considers dosimetric information from the treatment planning system (TPS) as well as temporal considerations such as the blood flow during treatment, was then used to simulate the radiation dose delivered to circulating blood.^13^

For this purpose, gantry angle‐wise dose distributions as well as a variety of treatment delivery parameters were exported from the TPS for every generated treatment plan. The latter included the irradiation time for each gantry angle as well as the energy switching time between gantry angles, determined via a model which has been validated previously.^22^, ^23^ The blood dose simulation workflow was constructed so that the dose distribution delivered from each gantry angle was delivered over the course of the total delivery time of the control point, which was calculated as the sum of the spot irradiation times, spot scanning times, burst switching times, and energy layer switching times associated with the gantry angle in question. Between gantry angles, the beam was turned off for either the required energy layer switching time or for the time required to rotate the gantry to the subsequent gantry angle at a rate of one rotation per minute, whichever time was longer. The dynamic treatment delivery of PAT was therefore discretized via the simulation of treatment delivery from 144 gantry angles, in exact agreement with the dose calculations and optimizations performed in the treatment planning system itself. Subsequently to nominal simulations, PAT blood dose simulations were repeated assuming the same delivery time as the respective IMPT treatment plan in order to help clarify whether observed differences in blood dose values between IMPT and PAT were the result of the underlying dose distributions or differences in delivery time.

HEDOS simulations considered the default total of 100,000 blood particles and were performed at a temporal resolution of 0.05 seconds. This resulted in a total of 10902 time steps for the average IMPT delivery time of 545.1 seconds, 7830 time steps for the average PAT delivery time of 391.5 seconds, and 13758 time steps for the maximum delivery time of 687.9 seconds considered in this study. HEDOS simulations utilize blood compartment‐specific blood volumes and flow rates specified in International Commission on Radiological Protection (ICRP) Publication 89, with male and female patients being assigned different reference values.^24^ It is to be noted that the simulated blood particles served as arbitrary representations of blood volume. That is to say that, while lymphocyte counts and the total blood volume within the body are patient‐dependent, this solely impacts the amount of blood volume (or the number of lymphocytes) represented by each blood particle. A sensitivity analysis focusing on the number of simulated blood particles was therefore not necessitated.

### 2.3 Data evaluation

Data analysis and visualization were performed in Python. The Wilcoxon signed‐rank test was used to determine whether radiation dose metrics for target structures, organs‐at‐risk, and circulating blood particles during PAT were statistically significantly (*p <* 0.05) different from their IMPT counterparts, as well as to investigate the significance of differences in integral dose associated with the two treatment techniques. In addition, linear least‐squares regression was used to investigate a potential correlation between blood compartment doses and a variety of blood dose metrics. This included the mean dose over all simulated blood particles D_mean_ as well as D_90%_ and D_10%_—i.e., the minimum dose delivered to the 90% and 10% of blood particles receiving the highest dose, respectively. For this particular analysis, blood compartment and blood dose data for IMPT and PAT was analyzed together rather than separately, since the objective was to identify potential correlations between blood compartment dose values and blood dose metrics rather than differences in such correlations between IMPT and PAT.

## 3 RESULTS

For all patients, treatment plans, and scenarios, the CTV coverage (for all high‐risk and low‐risk target volumes) and the mean dose in the larynx and both parotid glands as well as the dose received by the 2% of structure volume receiving the highest dose (D_2%_) of the brainstem and the spinal cord are shown in Figure 2. During IMPT, the CTV volume receiving at least 95% of its prescription dose (V95%) was (99.2 ± 1.6)% in the nominal and (98.0 ± 2.6)% in the worst‐case scenario, while during PAT, it was significantly (*p <* 0.05) different, with values of (99.2 ± 1.6)% (*p =* 0.034) and (97.9 ± 3.5)% (*p =* 0.021), respectively. The dose delivered to the 2% of CTV volume receiving the highest dose (D2%) was not statistically significantly different in the nominal scenario (*p =* 0.075) but was significantly higher (*p =* 0.007) in the worst‐case scenario of IMPT ((71.7 ± 9.1)Gy(RBE)) than in the worst‐case scenario of PAT ((70.8 ± 8.4)Gy(RBE)). PAT also significantly reduced the mean dose to the larynx ((34.4 ± 20.8)Gy(RBE) compared to (24.8 ± 17.6)Gy(RBE) in the nominal scenario (*p =* 0.008), (38.2 ± 20.0)Gy(RBE) compared to (28.0 ± 17.6)Gy(RBE) in the worst‐case scenario (0.008)), left parotid gland ((13.5 ± 4.5)Gy(RBE) compared to (9.6 ± 3.3)Gy(RBE) in the nominal scenario (*p =* 0.004), (18.4 ± 4.9)Gy(RBE) compared to (13.5 ± 4.0)Gy(RBE) in the worst‐case scenario (*p =* 0.004)), and the right parotid gland in the nominal scenario ((12.3 ± 6.9)Gy(RBE) compared to (9.1 ± 5.2)Gy(RBE) (*p =* 0.008)). PAT also significantly reduced D2% to the brainstem ((10.0 ± 9.1)Gy(RBE) compared to (4.6 ± 4.7)Gy(RBE) in the nominal scenario (*p =* 0.020), (13.9 ± 11.9)Gy(RBE) compared to (8.4 ± 7.7)Gy(RBE) in the worst‐case scenario (*p =* 0.020)) and the spinal cord ((14.1 ± 8.9)Gy(RBE) compared to (5.7 ± 3.4)Gy(RBE) in the nominal scenario (*p =* 0.008), (17.8 ± 9.2)Gy(RBE) compared to (9.4 ± 5.2)Gy(RBE) in the worst‐case scenario (p = 0.004)).

**FIGURE 2:**
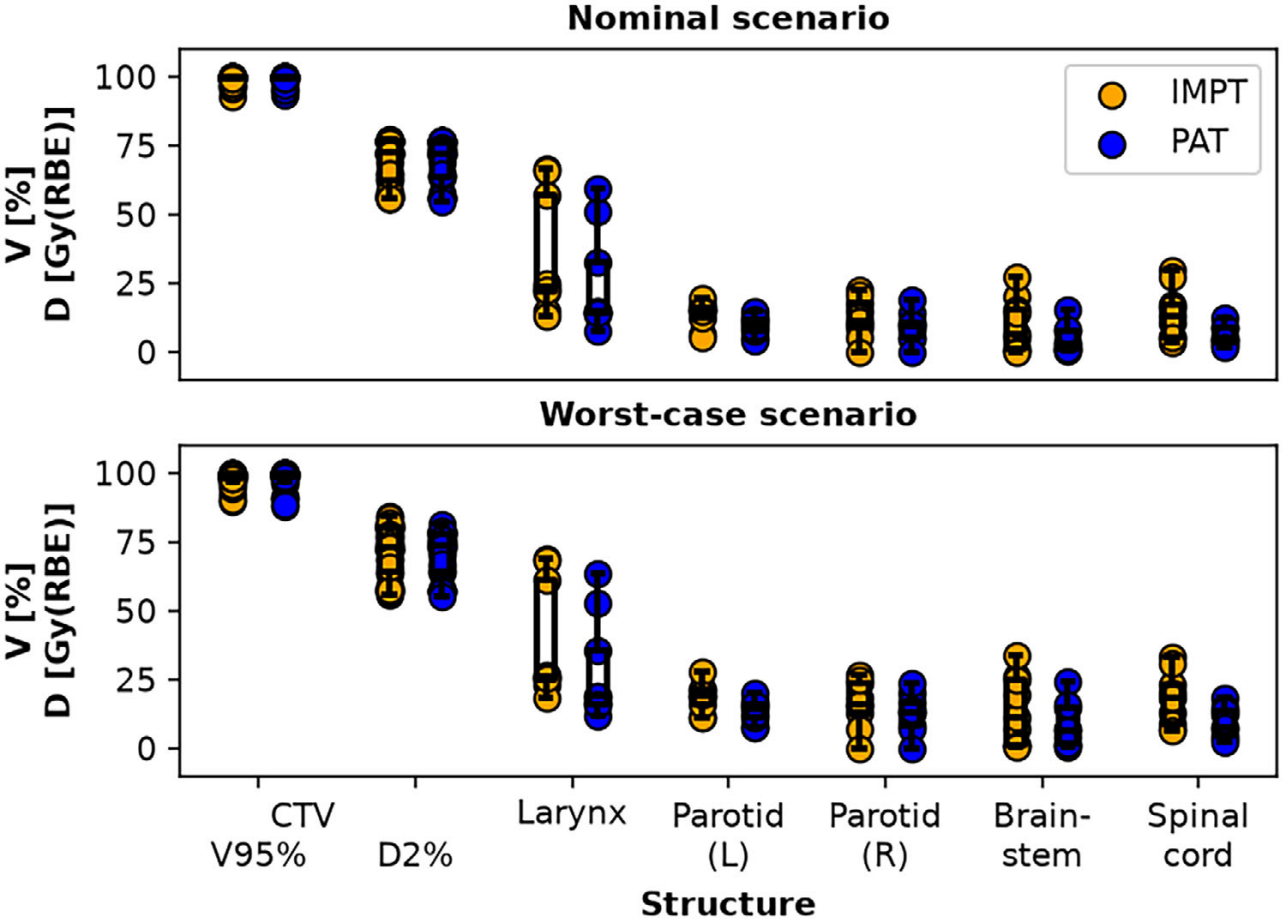
The clinical target volume (CTV) receiving at least 95% of the prescription dose (V95%), the dose delivered to the 2% of CTV volume receiving the highest dose (D2%), the mean dose in the larynx and both parotid glands as well as D2% of the brainstem and the spinal cord for intensity‐modulated proton therapy (IMPT; orange) and proton arc therapy (PAT; blue) treatment plans in the nominal (top) and worst‐case scenario (bottom). Every datapoint represents one structure, and CTV data includes all high‐risk and low‐risk target volumes.

The blood dose histograms generated for all patients and all IMPT and PAT treatment plans are shown in Figure 3. PAT consistently reduced a variety of blood dose metrics compared to IMPT.

**FIGURE 3:**
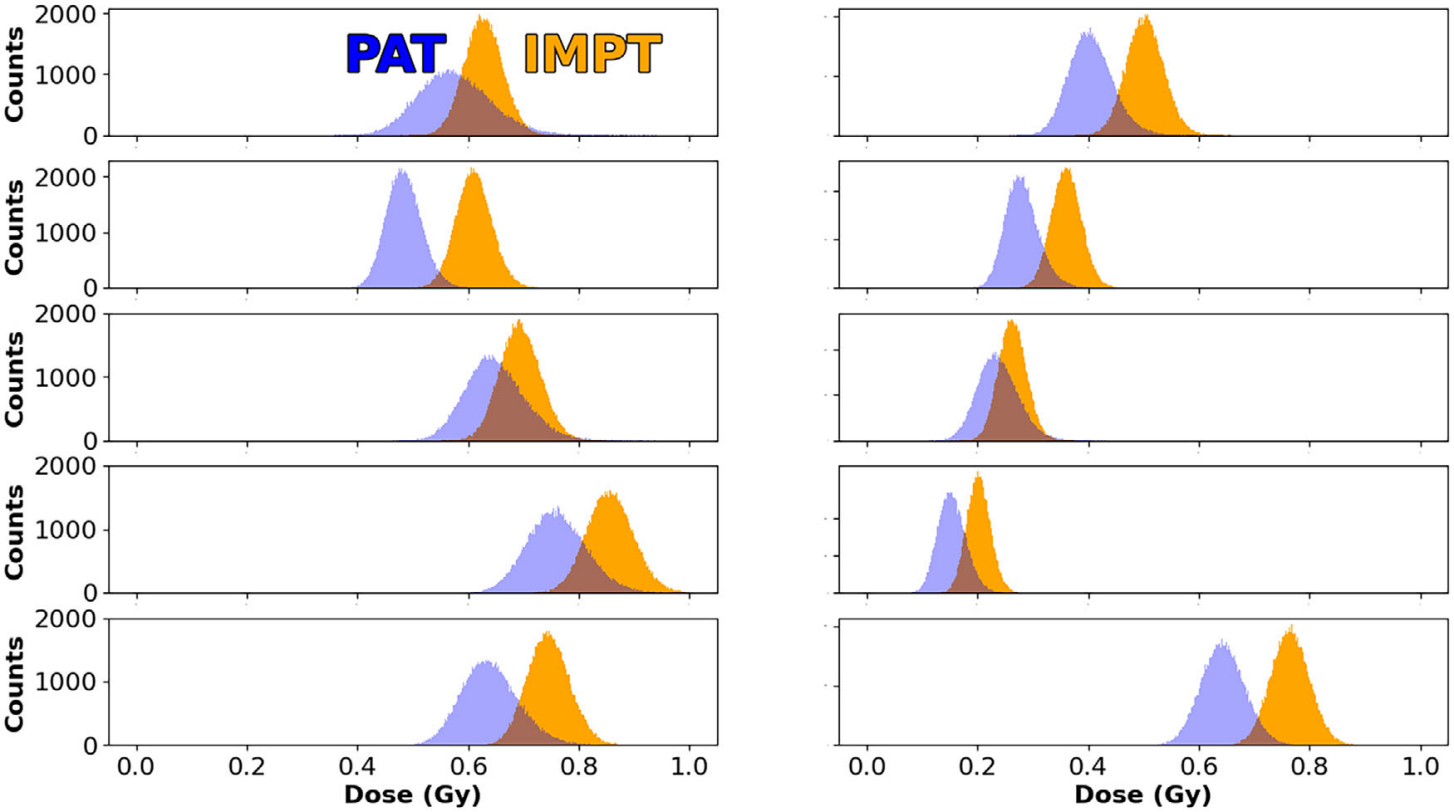
The blood dose histograms simulated for the delivery of all treatment fractions for intensity‐modulated proton therapy (IMPT; orange) and proton arc therapy (PAT; blue) for all ten patients. The y‐axis indicates the number of simulated blood particles (out of 100,000) which received the dose indicated on the x‐axis.

For all patients and IMPT as well as PAT, HD_95CTV‐BV_ and integral dose as well as a variety of different blood dose parameters are shown in Table 2. For IMPT treatment plans, blood dose metric distributions over all patients were (0.522 ± 0.204)Gy(RBE) for D_90%_, (0.563 ± 0.212)Gy(RBE) for D_mean_, and (0.605 ± 0.219)Gy(RBE) for D_10%_. The respective values for PAT were (0.430 ± 0.184)Gy(RBE) for D_90%_, (0.483 ± 0.195)Gy(RBE) for D_mean_, and (0.538 ± 0.207)Gy(RBE) for D_10%_. When PAT blood dose simulations were performed under the assumption of equal delivery time with IMPT, blood dose values were only marginally affected, being (0.428 ± 0.183)Gy(RBE) for D_90%_, (0.486 ± 0.196)Gy(RBE) for D_mean_, and (0.546 ± 0.209)Gy(RBE) for D_10%_. This was partially because, for delivery of the same treatment plan, changes in dose rate and therefore irradiation time change the way in which dose is distributed over the simulated blood particles, not e.g. the mean dose delivered to the blood particles. The Wilcoxon signed‐rank indicated a statistically significant (*p <* 0.05) difference between the integral dose and all evaluated blood dose metrics when comparing IMPT and PAT, with values of p = 0.002 for the integral dose and three dose metrics listed in Table 2. For both treatment modalities, a statistically significant correlation between HD_95CTV‐BV_ and all three evaluated blood dose metrics was likewise observed (*p =* 0.002 throughout).

**TABLE 2:** The 95% Hausdorff distance between clinical target volume and blood vessels (HD_95CTV‐BV_), total irradiation time (T_irrad_) and integral dose (D_int_) as well as a variety of blood dose metrics for the intensity‐modulated proton therapy (IMPT) and proton arc therapy (PAT) treatment plan of all patients. D_90%_, D_mean_, and D_10%_ refer to the minimum dose delivered to the 90% of blood particles receiving the highest dose, the mean dose to all simulated blood particles, and the minimum dose delivered to the 10% of blood particles receiving the highest dose, respectively, while V_0 Gy_ refers to the percentage of blood particles receiving no dose during the delivery of a single treatment fraction.

		IMPT						PAT					
Patient	HD95_CTV‐BV_ [cm]	T_irrad_ [s]	D_int_ [Gy(RBE)/L]	D_90%_ [Gy]	D_mean_ [Gy]	D_10%_ [Gy]	V_0 Gy_ [%]	T_irrad_ [s]	D_int_ [Gy(RBE)/L]	D_90%_ [Gy]	D_mean_ [Gy]	D_10%_ [Gy]	V_0 Gy_ [%]
**1**	5.4	576.5	0.768	0.584	0.628	0.672	0.000	234.8	0.676	0.496	0.576	0.661	0.064
**2**	4.5	548.3	0.801	0.570	0.610	0.652	0.000	639.0	0.730	0.445	0.484	0.525	0.000
**3**	7.0	630.8	0.632	0.648	0.694	0.741	0.000	358.1	0.588	0.584	0.649	0.718	0.000
**4**	5.8	526.5	0.490	0.804	0.857	0.911	0.000	382.5	0.445	0.694	0.760	0.829	0.000
**5**	6.5	618.0	0.576	0.697	0.745	0.795	0.000	410.3	0.500	0.577	0.640	0.708	0.000
**6**	6.5	687.9	0.631	0.460	0.503	0.546	0.000	532.3	0.518	0.356	0.405	0.456	0.001
**7**	6.9	525.0	0.572	0.327	0.361	0.395	0.000	433.9	0.477	0.244	0.281	0.320	0.018
**8**	6.6	409.1	0.458	0.232	0.264	0.296	0.000	215.6	0.421	0.193	0.237	0.284	2.446
**9**	8.6	453.2	0.330	0.175	0.202	0.229	0.145	283.4	0.272	0.123	0.154	0.186	4.502
**10**	5.0	475.3	0.417	0.721	0.765	0.810	0.000	425.2	0.401	0.594	0.644	0.694	0.000
**Mean** ** ± std dev**		545.1 ± 86.0	0.567 ± 0.141	0.522 ± 0.204	0.563 ± 0.212	0.605 ± 0.219	0.015 ± 0.046	391.5 ± 130.3	0.503 ± 0.128	0.430 ± 0.184	0.483 ± 0.195	0.538 ± 0.207	0.703 ± 1.539

Table 3 depicts linear least‐squares regression r^2^ and p values for D_90%_, D_mean_, and D_10%_ under consideration of the mean dose delivered to each considered HEDOS blood compartment. Please note that for this analysis, IMPT and PAT treatment plans were analyzed together rather than separately, since the objective was to identify potential correlations between blood compartment dose values and blood dose metrics rather than differences in such correlations between IMPT and PAT. All blood compartment in which a non‐negligible (i.e., an average dose of < 1 Gy(RBE) over all patients) was deposited exhibited a statistically significant (*p <* 0.05) correlation with all three of the aforementioned blood dose metrics, with structures with a particularly high blood volume and/or blood flow (i.e., the aorta, large arteries and veins, lungs, and skeletal muscle) exhibiting particularly high r^2^ values.

**TABLE 3:** Blood compartment information as well as the mean dose delivered to all evaluated HEDOS blood compartments and linear least‐squares regression r^2^ and p values for D_90%_, D_mean_, and D_10%_. Blood volume refers to the structure's blood volume relative to the body's total blood volume while blood flow refers to the percentage of blood transitioning from a given compartment to a different compartment per second. For blood compartments with sex‐specific blood volumes and/or blood flows, F and M indicate whether the respective value applies to female or male patients. For the mean dose to a given blood compartment, the mean value ± one standard deviation over all patients and treatment plans is indicated (i.e., IMPT and PAT treatment plans were analyzed simultaneously rather than separately).

				HEDOS metrics					
				D_90%_		D_mean_		D_10%_	
Compartment	Blood volume [%]	Blood flow [%]	D_mean_ [Gy(RBE)]	r^2^	p	r^2^	p	r^2^	p
**Aorta**	2.70	100.0	5.7 ± 3.4	0.879	<0.001	0.890	<0.001	0.900	<0.001
**Brain**	1.20	12.0	0.6 ± 0.5	0.168	0.073	0.180	0.063	0.192	0.053
**Fat**	8.50 (F) 5.00 (M)	8.5 (F) 5.0 (M)	7.5 ± 1.8	0.381	0.004	0.375	0.004	0.367	0.005
**Large arteries**	2.80	100.0	34.5 ± 19.2	0.941	<0.001	0.956	<0.001	0.965	<0.001
**Large veins**	14.00	92.5 (F) 93.5 (M)	13.7 ± 11.0	0.906	<0.001	0.907	<0.001	0.902	<0.001
**Lungs**	10.50	99.5 (F) 97.5 (M)	2.0 ± 1.4	0.907	<0.001	0.906	<0.001	0.900	<0.001
**Pulmonary artery**	0.50	99.5 (F) 97.5 (M)	0.0 ± 0.0	0.114	0.145	0.106	0.161	0.100	0.181
**Skeletal muscle**	10.50 (F) 14.00 (M)	12.0 (F) 17.0 (M)	11.5 ± 2.9	0.864	<0.001	0.872	<0.001	0.876	<0.001
**Superior vena cava**	1.50	32.4 (F) 32.7 (M)	0.1 ± 0.3	0.200	0.048	0.188	0.056	0.174	0.067
**Thyroid**	0.06	1.5	47.6 ± 22.2	0.663	<0.001	0.678	<0.001	0.691	<0.001

## 4 DISCUSSION

Compared to IMPT, proton arc therapy was associated with significantly lower integral body dose, organ‐at‐risk doses, and radiation dose delivery to circulating blood, including when the same delivery time was assumed for PAT as for IMPT. The ability of proton arc therapy to reduce the integral dose delivered to the patient has been documented previously, including for patients with head‐and‐neck cancer.^25^, ^26^, ^27^, ^28^ In addition, proton arc therapy has been reported to be associated with superior dose conformality to the target, further improving sparing of relevant structures in close proximity to the irradiated target(s), which may constitute large blood depositories.^1^, ^2^, ^3^, ^4^ IMPT did not reduce the percentage of blood particles receiving any radiation dose during delivery of a single treatment fraction. In light of considerable uncertainties in the definition of relevant dose threshold values for lymphocyte preservation, it was decided to evaluate the percentage of lymphocytes receiving any dose rather than the percentage of lymphocytes receiving a dose above a certain (to some extent artificial) threshold. However, based on determined blood particle dose‐volume histograms, it is expected that PAT consistently outperformed IMPT with respect to the number of blood particles receiving a dose above various different thresholds during the delivery of a single treatment fraction.

Determined blood dose metrics and the difference in values associated with IMPT and PAT depended on a variety of patient‐specific factors, including the prescription doses and volumes of all target (sub)structures and the tumor location. Amongst other considerations, the primary treatment site determined the tumor's distance to the delineated blood vessels, the dose to which exhibited a particularly strong correlation with simulated blood dose values. Particularly low simulated blood dose values were observed in the case of patient 8 and patient 9, which constituted the only case with a unilateral target and the case with the lowest radiotherapy prescription dose and a relatively small high‐risk target volume, respectively.

As discussed above, HEDOS blood dose simulations utilized a variety of patient‐specific variables, including patient sex. While patient‐specific total blood volume and cardiac output values were not available, the use of sex‐specific reference values is not expected to significantly impact this study's findings. Differences between the patient's true total blood volume and the reference value, for example, would solely have a small impact on the blood volume represented by one of the 100,000 blood particles included in a given simulation. For blood compartments which were not fully included in the CT FOV, treatment site‐specific blood flow values were also not available, resulting in blood flow in the relevant compartments being modeled via consideration of the general systemic circulation instead. This especially concerned major veins and arteries as well as skeletal muscle.

Rather than relying on the cumulative dose distribution delivered over all gantry angles, as is the norm during conventional radiotherapy treatment plan analysis, radiation dose delivery was simulated on a gantry angle‐wise basis. Energy layer‐ and especially proton pencil beam spot‐wise simulation of radiotherapy delivery would be associated with an enormous computational burden and was therefore considered computationally infeasible, especially for a considerable number of patients and treatment plans. However, while the simulation approach will affect some (but not all) of the determined blood dose metrics, it is not expected to have a considerable impact on this study's findings, which partially result from simulation approach‐independent considerations such as static blood compartment doses associated with PAT relative to IMPT, and efforts are now underway to eventually allow for at least exemplary blood dose simulations of dose distributions providing even greater detail of treatment delivery dynamics to confirm that this is the case.

This work focused on the radiation dose delivered to circulating blood, which only contains a fraction of the total lymphocyte pool.^29^, ^30^ Even so, irradiation of circulating blood was considered essential because radiation‐induced lymphocyte killing has been reported even in cases in which little lymphoid tissue is irradiated, and because estimates of radiation dose delivery to circulating blood have previously been shown to be correlated with severe radiation‐induced lymphopenia as well as poorer clinical outcomes, including in patients with head‐and‐neck cancer.^14^, ^15^, ^31^, ^32^, ^33^ In addition, the reported superior dose conformality of PAT is expected to simultaneously improve sparing of nearby lymphocyte repositories such as lymph nodes which are not included in the deliberated irradiated target structure(s).

## 5 CONCLUSION

Based on 10 patients with head‐and‐neck cancer, proton arc therapy was found to significantly (*p <* 0.05) reduce integral body dose, organ‐at‐risk doses, and the radiation dose delivered to circulating blood compared to IMPT. During delivery of a single treatment fraction, IMPT did not achieve a reduction in the percentage of irradiated (*D >* 0.0 Gy) blood particles. Proton arc therapy for head‐and‐neck cancer is therefore not expected to be associated with higher killing of circulating lymphocytes compared to conventional IMPT.

## AUTHOR CONTRIBUTIONS

ST contributed to project conception, data acquisition, analysis, and visualization, and the writing and editing of the manuscript. PL contributed to project conception, data acquisition and analysis, and the writing and editing of the manuscript. XC contributed to data acquisition and analysis and the writing and editing of the manuscript. CH and XD contributed to project conception, resource provision, funding acquisition, and the writing and editing of the manuscript.

## CONFLICT OF INTEREST STATEMENT

XD received honorariums from IBA and Elekta's speaker bureau outside the work presented here. XD received industrial research funding from IBA and Elekta outside this work.

## ETHICS STATEMENT

Not applicable. This retrospective study utilized previously generated, fully anonymized patient data for a dosimetric analysis. No prospective patient enrollment or intervention was performed.

## Data Availability

The CT datasets analyzed during the current study are publicly available in the “HNSCC‐3DCT‐RT” (DOI: DOI: 10.7937/K9/TCIA.2018.13upr2xf) as well as the “Head‐Neck‐PET‐CT” (DOI: 10.7937/K9/TCIA.2017.8oje5q00) datasets via The Cancer Imaging Archive as well as via the “HECKTOR 2025” grand challenge (https://hecktor25.grand‐challenge.org/dataset/). Derived data generated for the current study are available from the corresponding author upon reasonable request.
